# Meaning search as a mediator between post-traumatic stress symptoms and burnout in the long-term recovery phase after the 2023 Kahramanmaraş earthquakes

**DOI:** 10.3389/fpubh.2026.1781764

**Published:** 2026-05-12

**Authors:** Metin Çınaroğlu, Eda Yılmazer

**Affiliations:** 1 Faculty of Administrative and Social Science, Psychology Department, İstanbul Nişantaşı University, İstanbul, Türkiye; 2 Faculty of Social Science, Psychology Department, Beykoz University, İstanbul, Türkiye

**Keywords:** burnout, earthquake, long-term recovery, meaning search, mediation, post-traumatic stress symptoms

## Abstract

**Background:**

Large-scale earthquakes are associated with persistent psychological sequelae that extend beyond the acute post-disaster phase. While post-traumatic stress symptoms (PTSS) are well documented among disaster survivors, less is known about the psychological mechanisms linking PTSS to burnout during long-term recovery. The search for meaning following trauma represents a core existential process that may play a critical role in this association.

**Methods:**

This cross-sectional study examined adult survivors of the 6 February 2023 Kahramanmaraş earthquakes residing in Hatay, Türkiye (*N* = 419). Data were collected approximately 18–20 months post-disaster (November 2024–January 2025). Participants completed validated Turkish versions of the PTSD Checklist for DSM-5 (PCL-5), the Search for Meaning subscale of the Meaning in Life Questionnaire (MLQ), and the Shirom–Melamed Burnout Measure (SMBM). Mediation analyses were conducted using Hayes’ PROCESS macro (Model 4) with 5,000 bootstrap samples, controlling for sociodemographic variables, earthquake-related exposure, and psychiatric history/treatment.

**Results:**

Higher PTSS were significantly associated with higher burnout levels. PTSS were also positively associated with meaning search, which in turn was positively associated with burnout. Mediation analyses indicated a significant indirect effect of PTSS on burnout through meaning search, while the direct effect of PTSS on burnout remained significant, consistent with partial mediation. All effects remained significant after adjustment for relevant covariates.

**Conclusion:**

Findings suggest that meaning search may partially account for the association between long-term post-traumatic stress symptoms and burnout among earthquake survivors. Although searching for meaning is a natural response to trauma, prolonged or unresolved meaning search may contribute to emotional exhaustion during extended recovery periods. Interventions addressing existential concerns and facilitating adaptive meaning-making may help mitigate burnout among disaster survivors in the long-term recovery phase.

## Introduction

Large-scale natural disasters can impose lasting psychological burdens on survivors (1). Earthquakes in particular are often followed by elevated rates of post-traumatic stress disorder (PTSD), depression, and anxiety in affected communities (2). The twin Kahramanmaraş earthquakes of February 6, 2023 – among the most devastating in Türkiye’s history – exemplify this toll. Even 18 months post-event, many survivors remain displaced in temporary housing with ongoing disruptions to their social and professional lives (3). In such protracted recovery conditions, a substantial proportion of survivors report persistent post-traumatic stress symptoms (PTSS, (4)). Recent estimates indicate that over half of adults exposed to the 2023 earthquakes met PTSD criteria in the first year (5), and significant symptoms can endure long after the disaster. Importantly, chronic exposure to post-disaster stressors may also lead to burnout – a state of physical and emotional exhaustion typically studied in occupational contexts (6). Indeed, burnout has been observed among disaster survivors and responders in the long-term recovery phase (7). For example, professionals such as healthcare workers and teachers showed elevated burnout following major earthquakes (8). By definition, burnout is characterized by fatigue, disengagement, and cynicism resulting from prolonged stress and workload (9). The sustained hyperarousal and emotional strain associated with PTSD may accelerate burnout (10), as trauma survivors often experience ongoing physiological stress, social withdrawal, and negative affect that erode their emotional resources (11). Together, these findings underscore that *beyond acute trauma reactions*, survivors face risks of both PTSD and burnout as enduring consequences of large-scale disasters in the long-term recovery period.

Burnout has historically been conceptualized within occupational contexts and has most often been studied among professionals exposed to chronic job-related stressors. However, contemporary conceptualizations increasingly describe burnout as a broader state of physical, emotional, and cognitive exhaustion resulting from prolonged exposure to persistent demands that exceed an individual’s available psychological resources. The Shirom–Melamed Burnout Measure (SMBM), used in the present study, conceptualizes burnout within the framework of Conservation of Resources theory and operationalizes burnout as a depletion of energetic resources rather than a strictly job-specific construct. From this perspective, burnout-like exhaustion may also arise in non-occupational contexts characterized by sustained stress exposure. Large-scale disasters represent such environments, as survivors often face prolonged displacement, housing instability, financial strain, and ongoing uncertainty during recovery periods. These chronic stressors can lead to persistent exhaustion and disengagement that resemble the burnout process described in occupational research. In this context, burnout is conceptualized as a broader stress-related depletion syndrome that is theoretically distinguishable from general psychological distress by its emphasis on energetic resource loss and chronic exhaustion rather than acute emotional symptoms alone.

The quest for meaning is a critical theoretical lens for understanding such long-term psychological adjustment (12). Traumatic events can fundamentally shake one’s core beliefs and life narrative, often prompting survivors to search for meaning in the aftermath of loss and chaos (13). Frankl’s (14) existential theory (logotherapy) posits that the will to find meaning is a primary human drive that becomes especially salient following trauma. A disaster can leave individuals confronting an “existential vacuum,” wherein previous sources of purpose are lost or called into question (15). Frankl (16) noted that a loss of meaning often underlies profound despair, whereas discovering new meaning in suffering is crucial for resilience. Contemporary research further refines this concept by distinguishing between the presence of meaning in life and the search for meaning (17). The search for meaning refers to an active, ongoing effort to find purpose or significance in one’s life (18). Notably, survivors with high PTSS frequently report grappling with existential questions – “Why did this happen? What does my life mean now?” – indicating that trauma can spur an intensified search for meaning (19). However, the psychological impact of this search appears double-edged. On one hand, engaging in meaning search may be a necessary part of post-traumatic growth and recovery; for instance, seeking meaning has been linked to positive outcomes like posttraumatic growth in disaster survivors under certain conditions (20). On the other hand, a protracted, unresolved search for meaning can be psychologically taxing (21). Studies have found that a heightened search for meaning is often associated with greater distress – including symptoms of depression, anxiety, and grief – especially when individuals struggle to attain a sense of closure or purpose (22). The pursuit of meaning can generate tension and frustration if answers remain elusive. In the context of burnout, an existential perspective suggests that lacking a sense of meaning in one’s life or work can fuel emotional exhaustion and cynicism (23). Burnout has even been described as a state of “existential emptiness” resulting from a thwarted search for meaning in one’s endeavors. Thus, survivors who continue to search for meaning long after a trauma may be more vulnerable to burnout if their quest remains unfulfilled (24). Conversely, those who successfully find meaning in the trauma might experience better psychological outcomes, highlighting the complex role of meaning-making in post-disaster adaptation.

The present study builds on theoretical models of trauma adaptation suggesting that cognitive and existential processing processes may help explain how persistent trauma symptoms translate into longer-term psychological outcomes. Survivors experiencing elevated post-traumatic stress symptoms often engage in ongoing efforts to understand and integrate the traumatic event into their life narrative. This search for meaning may represent an important psychological mechanism linking trauma-related distress to broader exhaustion outcomes. When individuals struggle to resolve existential questions raised by traumatic events, the resulting cognitive and emotional strain may contribute to persistent fatigue, disengagement, and burnout. From this perspective, the search for meaning may function as an intermediary process through which post-traumatic stress symptoms influence burnout during prolonged disaster recovery.

Despite growing recognition of these existential processes, there is a distinct gap in the literature regarding how meaning search contributes to long-term post-disaster outcomes. Most research on disaster mental health has focused on prevalence of PTSD and depression or on resilience factors. Far less is known about the mechanisms that link trauma exposure to syndromes like burnout in the protracted recovery phase. In particular, the potential mediating role of survivors’ ongoing search for meaning remains underexplored. Addressing this gap is important because it can elucidate why some individuals with high PTSD symptoms also experience severe burnout over a year after the event. The present study is the first, to our knowledge, to examine meaning search as a mediator between PTSS and burnout in survivors of the 2023 Kahramanmaraş earthquakes during the long-term recovery period. Approximately 18–20 months post-disaster, we assessed adult survivors for PTSD symptom severity, degree of search for meaning in life, and burnout symptoms. We hypothesized that post-traumatic stress symptoms would be positively associated with burnout, and that this relationship would be mediated by the search for meaning. In particular, survivors with more severe PTSS were expected to report a heightened search for meaning, which in turn would be related to higher burnout levels. By testing this mediation model, our aim was to shed light on an underlying psychological mechanism – the struggle to find meaning – that may partly explain how long-term trauma distress translates into chronic exhaustion. Clarifying this mechanism can inform targeted interventions in the aftermath of large-scale disasters, ultimately helping survivors not only recover from PTSD symptoms but also reconnect with a sense of meaning to prevent burnout in the long run.

## Methods

### Study design

This study employed a single-site, cross-sectional observational design to investigate psychological outcomes during the long-term recovery phase following the 6 February 2023 Kahramanmaraş earthquakes. Data were collected in Hatay (one of the hardest-hit provinces) approximately 18–20 months post-disaster, between 1 November 2024 and 31 January 2025. This timing allowed assessment of persistent post-traumatic symptomatology and burnout beyond the acute aftermath. The primary objective was to test a mediation model in which PTSS served as the independent variable, meaning search was the mediating variable, and burnout was the outcome. All study measures were administered in their validated Turkish versions: PCL-5, the Meaning in Life Questionnaire (MLQ – Search subscale), and SMBM. A brief sociodemographic and earthquake exposure questionnaire was also included for sample characterization.

### Participants

A total of 419 adult survivors of the earthquakes participated. All were residents of Hatay province and had direct exposure to the February 2023 earthquakes. Inclusion criteria were: (1) age 18 years or older; (2) direct experience of the earthquake’s impact (such as life-threatening events, physical injury, bereavement of a close relative, displacement, or severe property damage); and (3) ability to provide informed consent. No exclusions were made on the basis of pre-existing psychiatric diagnoses, use of psychotropic medication, or current psychological/psychiatric treatment, in order to enhance ecological validity. The only exclusion criteria were refusal or inability to give consent and survey responses with substantial missing data on primary study variables.

### Procedure

Local clinical psychology graduate students in Hatay recruited participants through structured community outreach conducted in temporary housing areas (container cities), neighborhood community centers, and public gathering locations (e.g., local municipal service points and social support units). These settings reflect the primary living and support environments for earthquake survivors during the long-term recovery phase. Written informed consent was obtained in face-to-face sessions. After giving consent, each participant completed the battery of questionnaires via a tablet-administered Google Forms survey. The survey was administered in person by the researchers, who provided standardized instructions and clarification as needed. This face-to-face administration ensured completeness of data and uniform understanding of the questions.

## Measures

### Post-traumatic stress symptoms

PTSS were measured using PCL-5, a self-report instrument developed by Weathers (25) at the U.S. National Center for PTSD to assess PTSD symptom severity in accordance with DSM-5 diagnostic criteria. The PCL-5 consists of 20 items evaluating symptoms experienced during the past month, rated on a 5-point Likert scale ranging from 0 (*not at all*) to 4 (*extremely*), yielding a total severity score between 0 and 80, with higher scores indicating greater PTSD symptom severity. The Turkish version of the PCL-5 was validated by Boysan et al. (26) in clinical and community samples, demonstrating excellent internal consistency, sound construct validity, and a factor structure consistent with DSM-5 symptom clusters. In that validation study, the total scale showed high internal reliability (Cronbach’s *α* ≈ 0.93) and good convergent validity with trauma-related symptom measures.

### Meaning search

Meaning search was assessed using the Search for Meaning subscale of MLQ, originally developed by Steger et al. (17) to evaluate individuals’ active pursuit of meaning and purpose in life. The MLQ consists of 10 items divided into two distinct subscales—Presence of Meaning and Search for Meaning—each comprising five items. Items are rated on a 7-point Likert scale ranging from 1 (*absolutely untrue*) to 7 (*absolutely true*), with higher scores indicating greater engagement in meaning-seeking processes. The Turkish adaptation of the MLQ was conducted by Demirdağ and Kalafat (27), who demonstrated good construct validity, linguistic equivalence, and reliability in a university sample. In their validation study, internal consistency coefficients (Cronbach’s *α*) ranged from 0.81 to 0.85 for the two MLQ subscales, with acceptable test–retest reliability.

### Burnout

Burnout was assessed using SMBM, a multidimensional self-report instrument developed by Shirom and Melamed (28) to assess burnout as a depletion of physical, emotional, and cognitive energy resources, grounded in the Conservation of Resources theory. The SMBM consists of 14 items covering three core dimensions: physical fatigue, emotional exhaustion, and cognitive weariness. Items are rated on a 5-point Likert scale ranging from 1 (*almost never*) to 5 (*almost always*). In the present study, the mean score of all items was used as the overall burnout indicator, with higher scores reflecting greater burnout severity (possible range: 1–5). The Turkish adaptation of the SMBM was conducted by Ülbeği and İplik (29), who demonstrated good construct validity, criterion-related validity, and reliability in a sample of academic and administrative staff. Confirmatory factor analysis supported the original three-factor structure, and internal consistency coefficients for the subscales ranged from α = 0.66 to 0.81, indicating acceptable reliability.

### Statistical analysis

All analyses were performed using IBM SPSS Statistics (Version 28). Data screening was first conducted to ensure completeness of responses and to check for outliers or non-normal distributions. Descriptive statistics (mean, standard deviation, and range) were calculated for all key variables, and internal consistency (Cronbach’s alpha) was computed for each scale. Bivariate relationships among PTSS, meaning search, and burnout were examined with Pearson product–moment correlation coefficients. To test the hypothesized mediation, we used Hayes’ PROCESS macro (Model 4) in SPSS. In the mediation model, the total PCL-5 score (PTSS) was entered as the independent variable (X), the MLQ–Search score as the mediator (M), and the SMBM burnout score as the dependent variable (Y). Age, gender, level of housing damage, displacement status, earthquake-related bereavement, and indicators of psychiatric history or current treatment were included as covariates in the model to control for their potential influence. The indirect effect of PTSS on burnout via meaning search was estimated using bootstrap resampling (5,000 samples) to generate bias-corrected 95% confidence intervals. An indirect effect was deemed statistically significant if its 95% bootstrap confidence interval did not include zero. All significance tests were two-tailed with an alpha level of 0.05. Additional regression diagnostics were conducted to examine potential violations of model assumptions. Multicollinearity was assessed using variance inflation factors (VIF), and homoscedasticity was evaluated through visual inspection of residual plots. The proportion of explained variance (R^2^) was also calculated for the mediator and outcome regression models to assess the explanatory power of the model.

## Results

### Descriptive statistics

Sample demographic and exposure characteristics are summarized in Table 1. Participants had a mean age of 36.8 years (SD = 11.4), and 58.0% identified as female (Table 1). The majority of the sample (68.5%) experienced severe damage to their housing, 60.1% were displaced from their homes after the earthquakes, and 32.9% reported the loss of a close family member in the disaster (Table 1). Regarding mental health history, 22.9% of participants indicated a prior psychiatric diagnosis, and 26.7% were receiving psychotropic medication or psychotherapy at the time of the survey (Table 1).

**Table 1:** Sample characteristics (demographics and earthquake exposure).

Characteristic	*n* (%) or Mean ± SD
Age (years)	36.8 ± 11.4
Gender	
Female	243 (58.0)
Male	176 (42.0)
Education (≥ high school)	301 (71.8)
Currently employed	214 (51.1)
Earthquake exposure	
Severe housing damage	287 (68.5)
Displacement after earthquake	252 (60.1)
Bereavement (loss of close relative)	138 (32.9)
Mental health status	
Prior psychiatric diagnosis	96 (22.9)
Current medication or psychotherapy	112 (26.7)

Descriptive statistics for the key psychological measures and their internal reliabilities are presented in Table 2. The mean PCL-5 score (PTSS severity) was 34.7 (SD = 15.8) out of a possible 0–80 range. The MLQ–Search (meaning search) had a mean of 21.9 (SD = 6.7) on a 5–35 possible range. The average burnout level (SMBM mean score) was 3.21 (SD = 0.91) on a scale from 1 to 5. All three scales demonstrated high internal consistency in this sample: Cronbach’s *α* was 0.94 for the PCL-5, 0.85 for the MLQ–Search subscale, and 0.92 for the SMBM (Table 2). These values indicate that the measures were reliable in our study.

**Table 2:** Descriptive statistics for PTSS, meaning search, and burnout, and Cronbach’s α for each scale.

Variable	Mean ± SD	Min–Max (possible)	Cronbach’s α
PCL-5 (0–80)	34.7 ± 15.8	0–80	0.94
MLQ–Search (5–35)	21.9 ± 6.7	5–35	0.85
SMBM (mean score; 1–5)	3.21 ± 0.91	1–5	0.92

## Correlations

Pearson correlation coefficients among PTSS, meaning search, and burnout are shown in Table 3. All bivariate associations were statistically significant. Higher post-traumatic stress symptom levels were moderately associated with higher burnout, *r* = 0.58, *p* < 0.001. PTSS was also positively correlated with meaning search, *r* = 0.34, *p* < 0.001. In addition, meaning search had a positive correlation with burnout, *r* = 0.29, *p* < 0.001 (Table 3). Thus, individuals with more severe PTSS tended to report greater engagement in searching for meaning, and both higher PTSS and greater meaning search were associated with higher burnout levels.

**Table 3:** Pearson correlation matrix among PTSS, meaning search, and burnout.

Variable	1	2	3
1. Post-traumatic stress symptoms (PCL-5)	—		
2. Meaning search (MLQ–Search)	0.34**	—	
3. Burnout (SMBM)	0.58**	0.29**	—

## Mediation analysis

**Figure 1:**
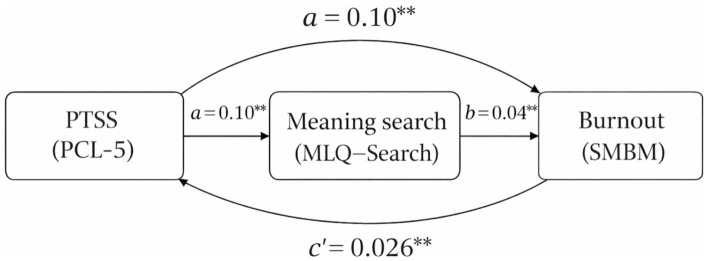
Mediation model illustrating the relationship between post-traumatic stress symptoms (PTSS), meaning search, and burnout. Unstandardized regression coefficients are displayed for each path.

The mediation analysis results are summarized in Tables 4, 5. In the total effect model (without the mediator), PTSS had a significant positive association with burnout, *B* = 0.030, *SE* = 0.003, *t* = 10.00, *p* < 0.001, 95% CI [0.024, 0.036] (Tables 4, 5). This indicates that, overall, higher PTSS predicted higher burnout scores. After including meaning search as a mediator and controlling for covariates, the direct effect of PTSS on burnout remained significant, *B* = 0.026, *SE* = 0.003, *t* = 8.67, *p* < 0.001, 95% CI [0.020, 0.032] (Tables 4, 5). Importantly, PTSS was a significant positive predictor of the mediator (meaning search), *B* = 0.10, *SE* = 0.02, *t* = 5.00, *p* < 0.001, 95% CI [0.06, 0.14] (path *a* in Tables 4, 5). In turn, meaning search was a significant positive predictor of burnout when controlling for PTSS, *B* = 0.040, *SE* = 0.010, *t* = 4.00, *p* < 0.001, 95% CI [0.020, 0.060] (path *b* in Tables 4, 5). The indirect effect of PTSS on burnout via meaning search was *B* = 0.004 with a bootstrapped standard error of 0.001. The 95% bootstrap confidence interval for this indirect effect was [0.002, 0.007], as shown in Tables 4, 5. Because this interval does not include zero, the indirect effect is statistically significant. In other words, there was a small but significant mediated effect such that higher PTSS contributed to higher burnout partly through an increase in meaning search. The regression model predicting the mediator (meaning search) accounted for a modest proportion of variance (R^2^ ≈ 0.12). The final regression model predicting burnout explained a larger proportion of variance (R^2^ ≈ 0.36). Diagnostic checks indicated no evidence of problematic multicollinearity among predictors (all VIF values < 2), and inspection of standardized residual plots did not suggest violations of homoscedasticity assumptions. These diagnostics support the robustness of the regression-based mediation analysis. Figure 1 illustrates the mediation model linking PTSS, meaning search, and burnout, with unstandardized coefficients for each path. All reported effects remained significant after accounting for the demographic and exposure covariates included in the model. The mediation model remained statistically significant after adjusting for prior psychiatric diagnosis and current treatment status.

**Table 4:** Regression models, mediation analysis results (regression coefficients for each path and indirect effect with 5,000-bootstrap CI).

Outcome (model)	Predictor	*B*	*SE*	*t*	*p*	95% CI
M: MLQ–Search	PTSS *(a)*	0.10	0.02	5.00	<0.001	[0.06, 0.14]
Y: SMBM	PTSS *(c)*	0.030	0.003	10.00	<0.001	[0.024, 0.036]
Y: SMBM	PTSS *(c′)*	0.026	0.003	8.67	<0.001	[0.020, 0.032]
	MLQ–Search *(b)*	0.040	0.010	4.00	<0.001	[0.020, 0.060]

**Table 5:** Effects summary.

Effect	Estimate	Boot SE	95% Boot CI
Total effect (c)	0.030	0.003	[0.024, 0.036]
Direct effect (c′)	0.026	0.003	[0.020, 0.032]
Indirect effect (a × b)	0.004	0.001	[0.002, 0.007]

Indirect effects are based on 5,000 bootstrap samples. The indirect effect is significant when the 95% bootstrap CI does not include zero.

Figure 1 presents the mediation model illustrating the direct and indirect associations between post-traumatic stress symptoms, meaning search, and burnout. Unstandardized regression coefficients are shown for each path, and all depicted effects are statistically significant (*p* < 0.01).

## Discussion

This study examined the interplay between post-traumatic stress, the search for meaning, and burnout in survivors approximately 18–20 months after the 2023 Kahramanmaraş earthquakes. Consistent with expectations, we found that higher levels of PTSS were significantly associated with higher burnout in the long-term recovery phase. Furthermore, our mediation analysis supported the hypothesis that meaning search serves as a significant intervening variable in this relationship. Survivors reporting more severe PTSD symptoms also tended to report a greater active search for meaning in life, and this heightened meaning search partially mediated the link between PTSS and burnout. In practical terms, this suggests that individuals who are struggling with intense trauma symptoms may engage in an ongoing quest to make sense of their experience, and this unresolved quest is associated with greater exhaustion, fatigue, and disengagement (burnout). It is noteworthy that the mediation effect, while statistically significant, was partial – the direct relationship between PTSS and burnout remained robust. Although the indirect effect was statistically significant, its magnitude was relatively small. This finding suggests that the search for meaning represents only one of several psychological mechanisms linking post-traumatic stress symptoms to burnout in disaster survivors. Given the complex and multifactorial nature of post-disaster psychological adjustment, it is expected that no single mediator would account for a large proportion of the relationship between trauma symptoms and longer-term exhaustion outcomes. Factors such as ongoing environmental stressors, social support, coping strategies, and economic hardship likely contribute simultaneously to burnout during the recovery period. Therefore, the present findings should be interpreted as identifying a modest but theoretically meaningful pathway through which trauma-related distress may translate into emotional exhaustion. This indicates that meaning search explains part of the PTSD–burnout connection, but other pathways (e.g., chronic physiological arousal or social factors) likely also contribute. Nonetheless, the identification of meaning search as a mediator provides novel insight into the psychological mechanism linking trauma and long-term burnout. Given the cross-sectional design, the mediation findings should be interpreted as statistical associations rather than evidence of causal pathways, and the directionality of effects cannot be definitively established.

The finding that survivors with elevated PTSD symptoms engage more in meaning search aligns with theoretical models of trauma and meaning-making (30). Traumatic events often violate core beliefs and life assumptions, prompting survivors to question “why” the event occurred and “what now” in its aftermath. Our results confirm that even 1.5 years post-disaster, many survivors are still actively grappling with these existential questions. This extended meaning search likely reflects ongoing attempts at cognitive processing and sense-making of the earthquake experience (31). Crucially, however, our data suggest that this search for meaning has a double-edged impact. Rather than alleviating distress, a strong search for meaning was associated with *higher* burnout when controlling for PTSD severity. In other words, survivors who remain preoccupied with finding meaning in the trauma tend to feel more emotionally exhausted and cynical. This pattern resonates with emerging research showing that the process of searching for meaning can be stressful and fraught with tension, especially if one has not yet found satisfying answers (32). The pursuit of meaning may keep survivors ruminating on the trauma and its consequences, which can deplete emotional resources and perpetuate stress. Indeed, prior studies have reported mixed effects of meaning search on mental health, with some finding that an intense search correlates with greater psychological distress (33). Our results extend this literature by linking meaning search to burnout, suggesting that an unfulfilled existential quest might manifest in the classic burnout symptoms of fatigue, inefficacy, and detachment. From an existential perspective, this makes sense: burnout has been conceptualized as a state of “existential vacuum” or emptiness that can arise when one’s work or life loses meaning. Survivors in our study who are still seeking meaning may be those who *feel* a lack of meaning (since they have not yet found it), which could leave them especially vulnerable to feeling drained and demoralized.

It is important to emphasize that searching for meaning is not inherently maladaptive (34). The mediation we observed was partial and small in magnitude, indicating that meaning search is only one piece of a larger puzzle. In fact, engaging in meaning search can be an integral part of positive adaptation *if* it eventually leads to meaning found. The literature on posttraumatic growth (PTG) suggests that those who grapple with existential questions sometimes emerge with a deeper sense of purpose or appreciation for life (35). For example, a study of natural disaster survivors found that the search for meaning was positively associated with posttraumatic growth and considered “an important part of recovery” (36). Likewise, a recent investigation of trauma narratives reported that the ability to find meaning in a traumatic event was linked to greater posttraumatic growth and well-being (37). In our sample, we did not measure *presence* of meaning or successful meaning-making outcomes; thus, “meaning search” as captured here likely represents an ongoing, unresolved process. The absence of an association with reduced PTSD symptoms underscores that merely searching, without finding resolution, does not mitigate trauma-related distress – a pattern consistent with other research. In fact, meaning-making efforts show benefits primarily when they resolve the discrepancy between the traumatic experience and one’s global beliefs. Therefore, our findings can be interpreted as highlighting a potential risk of prolonged, unresolved meaning search: survivors still struggling to make meaning 18 months post-trauma are at risk of worse adjustment (higher burnout). This does not negate the possibility that those efforts could eventually bear fruit in the form of newfound meaning or growth; rather, it suggests a window of intervention, wherein guided support to facilitate meaning-making might prevent the search from devolving into chronic exhaustion.

The present findings contribute to the literature by highlighting the long-term recovery phase after a major disaster. Most research and interventions tend to concentrate on the acute aftermath (weeks or months post-disaster). By 18–20 months post-event, survivors are no longer in crisis mode, but our findings show they may still be far from fully recovered. In fact, the persistence of high PTSD and burnout symptoms in this sample underscores that the psychological impact of the earthquakes has been enduring. This is in line with recent evidence that PTSD can remain prevalent and impairing well beyond the first year after trauma (38). We found that even at this extended time point, survivors continue to wrestle with the meaning of what happened. The long-term context is important because many external supports (e.g., crisis counseling, acute aid) have tapered off by this stage. Yet survivors are dealing with secondary stressors such as rebuilding homes, financial strain, and ongoing displacement, which can compound their mental health challenges. Our results emphasize the value of examining these chronic adaptation processes. The mediation by meaning search suggests that even after the passage of time, psychological and existential processes are actively shaping outcomes. In other words, how survivors cognitively and emotionally process the trauma during the long-term recovery can influence whether they succumb to burnout or maintain better well-being. This finding aligns with the broader trauma literature which highlights that recovery is an ongoing, dynamic process, where factors like coping strategies, appraisals, and meaning-making play evolving roles over time (39, 40). By shedding light on survivors’ inner struggle for meaning 18–20 months after the disaster, our study contributes to a more nuanced understanding of long-term resilience and vulnerability. It underlines that “recovering” from a disaster is not just about symptom reduction, but also about reconstructing a coherent life narrative and purpose – a task that, if left incomplete, may have mental health consequences.

### Clinical and practical implications

These findings carry several implications for post-disaster mental health interventions. First, the robust association between PTSS and burnout indicates that practitioners should monitor burnout symptoms (such as chronic fatigue, loss of enthusiasm, and detachment) in trauma-exposed populations, and not only traditional PTSD or depression symptoms. Burnout in survivors may manifest as a loss of drive or hope in rebuilding their lives, which can impede recovery efforts. Our results specifically highlight unresolved meaning search as a therapeutic target. In practice, this means that interventions fostering meaning-making could be beneficial. Therapists and counselors working with earthquake survivors might incorporate meaning-centered techniques – for example, encouraging survivors to explore the personal significance of their survival, to memorialize losses, or to identify values and goals moving forward. Approaches grounded in logotherapy (41) or existential psychotherapy (42) are particularly relevant here. By helping individuals *resolve* their search for meaning – that is, to come to terms with “why” the disaster happened (even if the answer is finding meaning *despite* senselessness) or to discover new purpose in the aftermath – we may reduce the psychological strain that fuels burnout. There is evidence that bolstering the sense of meaning in life can promote better mental health outcomes. A recent systematic review found that meaning in life has a consistent negative association with PTSD symptoms and called for leveraging meaning as a focus in trauma interventions (43). This suggests that integrating meaning-based interventions (such as narrative therapies, group meaning-making workshops, or spiritual/existential support services) into long-term disaster recovery programs could mitigate both PTSD and burnout. For instance, community support groups could be facilitated for survivors to share their stories and derive meaning collectively, or psychotherapists could use techniques like *benefit-finding* and *values clarification* to help individuals reinterpret their experience in a way that restores a sense of purpose. By addressing the existential dimension of trauma, such interventions might not only reduce distress but also rekindle engagement and hope, thereby protecting against the demoralization and exhaustion of burnout. Ultimately, our findings reinforce the view that mental health recovery after disaster should encompass meaning-centered care, attending to the human need for meaning and purpose as a component of healing.

### Limitations

Several limitations of this study should be acknowledged when interpreting the results. First, the design was cross-sectional, capturing survivors at one time point nearly 20 months post-disaster. This limits our ability to draw causal conclusions about the directionality of relationships. While we theorized that PTSD symptoms lead to increased meaning search which then contributes to burnout, the opposite direction or bidirectional influences are also plausible. It is possible, for example, that individuals experiencing severe burnout become more prone to ruminate on existential issues (i.e., questioning life’s meaning when feeling exhausted and ineffective). Longitudinal data are needed to establish temporal order – whether early post-traumatic stress predicts subsequent meaning search and burnout, or if those with high burnout later report more PTSS – and to test the stability of the mediation over time. Second, the sample was drawn from a specific population of adult survivors in Hatay, one of the regions most severely affected by the earthquakes, using community outreach and convenience sampling. While this approach was necessary given the logistical and ethical constraints of conducting research in a post-disaster context, it introduces potential sampling biases. Individuals who agreed to participate may differ systematically from those who did not participate, for example in terms of psychological distress levels, availability of time, access to community networks, or willingness to engage with research activities. As a result, the sample may overrepresent survivors who were more accessible through community outreach channels or more willing to share their experiences. These factors limit the representativeness of the sample and restrict the generalizability of the findings to the broader population of earthquake survivors. Therefore, the observed associations among PTSS, meaning search, and burnout should be interpreted cautiously and replicated in more diverse and systematically recruited samples in future research. Cultural and community contexts can influence both how people experience meaning in life and how they report burnout. Caution should be taken in extending these findings to other disaster settings, unimpacted regions, or different cultural groups without additional research. Relatedly, the majority of our participants were living in a context of ongoing socio-economic hardship after the earthquake; results might differ in a context with more robust recovery infrastructure or among survivors who have returned to normal living conditions. Third, all measures were self-reported, raising concerns about common method variance and potential response biases. Participants’ reports of PTSS, meaning search, and burnout may have been influenced by subjective factors such as current mood or social desirability. However, we used well-validated instruments with good psychometric properties, and the distinct pattern of mediation (which is less likely to be an artifact of common method bias) offers some confidence in the relationships observed. Nonetheless, future studies could include multi-method assessments (e.g., clinical interviews for PTSD, informant reports, or physiological measures of stress) to corroborate self-report data. Another limitation is that we focused only on the search for meaning dimension and did not directly measure the presence of meaning in life. This was intentional given our interest in the restless aspect of meaning-making, but it means we cannot determine how many participants had successfully found meaning or how the presence of meaning might buffer burnout. Including both meaning-search and meaning-presence in future research would provide a more complete picture of the role of meaning in recovery. Finally, while we controlled for several demographic and exposure-related covariates, there may be unmeasured confounders (such as personality traits, social support, or physical health status) that influence the observed relationships. These factors should be explored in future research to isolate the unique contribution of meaning search.

## Future directions

Building on this study, future research should adopt longitudinal and prospective designs to track survivors over multiple time points post-disaster. A longitudinal approach would allow researchers to observe how meaning search and meaning-making evolve – for example, does the search for meaning peak several months after the trauma and then decline as people either find meaning or give up? And critically, do changes in meaning search predict subsequent changes in burnout or PTSD symptoms (or vice versa)? Such data could inform the optimal timing for interventions (e.g., perhaps meaning-focused interventions are most needed in the mid-recovery phase when the search is ongoing). Additionally, experimental and intervention studies are warranted. Given our findings, a logical next step would be to design or evaluate an intervention that explicitly targets *unresolved meaning search* among disaster survivors. This could involve a randomized controlled trial of a meaning-centered therapy module added to standard trauma-focused therapy, or a comparison of survivors who engage in a structured life-review and meaning-making program versus those who receive psychoeducation alone. Outcomes could include not only PTSD and burnout, but also positive indicators like posttraumatic growth and well-being. Researchers should also consider examining cultural factors in meaning-making. As some evidence suggests, Eastern and Western cultures may differ in how the search for meaning relates to well-being (with Eastern cultures viewing search and presence of meaning as more interconnected). Cross-cultural studies could explore whether the mediating role of meaning search holds universally or varies by cultural context, which can inform culturally sensitive interventions. Moreover, incorporating qualitative research could deepen our understanding of *what* survivors are searching for in terms of meaning (e.g., explanations, spiritual understanding, life priorities) and *why* some struggle to find it. Such insights can guide tailored approaches – for instance, if many survivors are seeking meaning through helping others, volunteer programs might be leveraged to facilitate meaning-making and reduce burnout simultaneously. Finally, expanding the scope beyond individual-level factors, future work could integrate how community and societal narratives (e.g., collective memorials, cultural framing of the disaster) assist or hinder survivors in finding meaning. In summary, future research should continue to unravel the complex role of meaning in trauma recovery, using rigorous designs to verify mediators and test interventions that promote meaningful resilience.

## Conclusion

In the aftermath of the 2023 Kahramanmaraş earthquakes, survivors continue to experience persistent post-traumatic stress symptoms alongside elevated levels of burnout during the long-term recovery phase. The present findings indicate that the ongoing search for meaning is significantly associated with this relationship and may represent a modest but meaningful psychological pathway linking trauma-related distress to emotional exhaustion. While meaning search is a natural and potentially adaptive response to trauma, prolonged and unresolved engagement in this process appears to be associated with greater burnout. These results highlight the importance of addressing existential and meaning-related processes in post-disaster mental health interventions. Supporting survivors in developing adaptive meaning-making may help reduce long-term psychological burden and promote more sustainable recovery.

## Data Availability

The datasets generated and/or analyzed during the current study are available from the corresponding author on reasonable request.

## References

[1] Saeed SA Gargano SP. Natural disasters and mental health. Int Rev Psychiatry. (2022) 34:16–25. doi: 10.1080/09540261.2022.2037524 35584023

[2] Çınaroğlu M Yılmazer E Noyan Ahlatcioglu E Ülker SV Hızlı Sayar G. Psychological impact of the 2023 Kahramanmaraş earthquakes: a systematic review and meta-analysis of PTSD, depression, and anxiety among Turkish adults. Front Public Health. (2025) 13:1664212. doi: 10.3389/fpubh.2025.1664212, 40933413 PMC12417135

[3] Toprak S Zulfikar AC Mutlu A Tugsal UM Nacaroglu E Karabulut S . The aftermath of 2023 Kahramanmaras earthquakes: evaluation of strong motion data, geotechnical, building, and infrastructure issues. Nat Hazards. (2025) 121:2155–92. doi: 10.1007/s11069-024-06890-w

[4] Taskin M Kemal BAS Ucak EF. Post-traumatic stress disorder (PTSD) in disaster survivors 1 year after the 2023 Kahramanmaras earthquake in Turkey. Euro J Trauma Dissoc. (2025) 9:100504. doi: 10.1016/j.ejtd.2025.100504, 38826717

[5] Kaya E Onal EI Fatih S Güler O. Prevalence and predictors of post-traumatic stress disorder among survivors of the 2023 earthquakes in Türkiye: the case of a temporary camp. Int J Disast Risk Reduc. (2024) 114:104976. doi: 10.1016/j.ijdrr.2024.104976

[6] Alavi SM Kia-Keating M Nerenberg C. Secondary traumatic stress and burnout in health care providers: a post-disaster study. Traumatology. (2023) 29:389–401. doi: 10.1037/trm0000418

[7] Kawashima Y Nishi D Noguchi H Usuki M Yamashita A Koido Y . Post-traumatic stress symptoms and burnout among medical rescue workers 4 years after the great East Japan earthquake: a longitudinal study. Disaster Med Public Health Prep. (2016) 10:848–53. doi: 10.1017/dmp.2016.83, 27188495

[8] Mattei A Fiasca F Mazzei M Necozione S Bianchini V. Stress and burnout in health-care workers after the 2009 L’Aquila earthquake: a cross-sectional observational study. Front Psych. (2017) 8:98. doi: 10.3389/fpsyt.2017.00098, 28659831 PMC5466955

[9] Maslach C Leiter MP. "Burnout". In: ed. Fink, G. Stress: Concepts, Cognition, Emotion, and Behavior. Amstedram: Academic Press (2016).

[10] Restauri N Sheridan AD. Burnout and posttraumatic stress disorder in the coronavirus disease 2019 (COVID-19) pandemic: intersection, impact, and interventions. J Am Coll Radiol. (2020) 17:921–6. doi: 10.1016/j.jacr.2020.05.021, 32479798 PMC7250786

[11] Carlson EB Dalenberg CJ. A conceptual framework for the impact of traumatic experiences. Trauma Violence Abuse. (2000) 1:4–28. doi: 10.1177/1524838000001001002

[12] Wong PT. The Human Quest for Meaning: Theories, Research, and Applications. London: Routledge (2013).

[13] Landsman IS. "Crises of meaning in trauma and loss". In: ed. Kauffman, J. Loss of the Assumptive World. London: Routledge (2013).

[14] Frankl VE. On the Theory and Therapy of mental Disorders: An Introduction to Logotherapy and Existential Analysis. London: Routledge. (2004).

[15] de Vries MFK. The Management of Loss: Humanity’s Existential Crises. London: Taylor & Francis (2025).

[16] Frankl V. Loss, recovery, and resilience. Strength Family Resilience. London (2015) 206.

[17] Steger MF Frazier P Oishi S Kaler M. The meaning in life questionnaire: assessing the presence of and search for meaning in life. J Couns Psychol. (2006) 53:80–93. doi: 10.1037/0022-0167.53.1.80

[18] Frankl VE. Search for Meaning. Milwaukee: Mount Mary College (1984).

[19] Aslan O Kabakçı ÖF Topuz İ Maden Yılmaz E. The search for meaning in earthquake survivors: an existentially positive psychology perspective. Arch Psychol Relig. (2025) 47:288–310. doi: 10.1177/00846724251329381

[20] Slade M Rennick-Egglestone S Blackie L Llewellyn-Beardsley J Franklin D Hui A . Post-traumatic growth in mental health recovery: qualitative study of narratives. BMJ Open. (2019) 9:e029342. doi: 10.1136/bmjopen-2019-029342, 31256037 PMC6609070

[21] Gordon R. Dying and Creating: A Search for Meaning. London: Routledge (2018).

[22] Wilt JA Stauner N Lindberg MJ Grubbs JB Exline JJ Pargament KI. Struggle with ultimate meaning: nuanced associations with search for meaning, presence of meaning, and mental health. J Posit Psychol. (2018) 13:240–51. doi: 10.1080/17439760.2017.1279208

[23] Engebretsen KM Bjorbækmo WS. Burned out or “just” depressed? An existential phenomenological exploration of burnout. J Eval Clin Pract. (2020) 26:439–46. doi: 10.1111/jep.13288, 31512347

[24] Galek K Flannelly KJ Greene PB Kudler T. Burnout, secondary traumatic stress, and social support. Pastor Psychol. (2011) 60:633–49. doi: 10.1007/s11089-011-0346-7

[25] Weathers FW. The Ptsd Checklist for dsm-5 (Pcl-5). London: National Center for PTSD (2013).

[26] Boysan M Guzel Ozdemir P Ozdemir O Selvi Y Yilmaz E Kaya N. Psychometric properties of the Turkish version of the PTSD checklist for diagnostic and statistical manual of mental disorders, (PCL-5). Psychiatry Clin Psychopharmacol. (2017) 27:300–10. doi: 10.1080/24750573.2017.1342769

[27] Demirdağ S Kalafat S. Meaning in life questionnaire (MLQ): the study of adaptation to Turkish, validity and reliability. J Facul Educ. (2015) 16:83–95. doi: 10.17679/iuefd.16250801

[28] Shirom A Melamed S. A comparison of the construct validity of two burnout measures in two groups of professionals. Int J Stress Manag. (2006) 13:176–200. doi: 10.1037/1072-5245.13.2.176

[29] Ülbeği İD İplik E. Shirom-Melamed tükenmişlik ölçeğinin güvenirlik ve geçerlik çalışması. Çağ Üniversitesi Sosyal Bilimler Dergisi. (2017) 14:19–30.

[30] Park CL. Meaning making following trauma. Front Psychol. (2022) 13:844891. doi: 10.3389/fpsyg.2022.844891, 35401307 PMC8984472

[31] Park CL. "Trauma and meaning making: converging conceptualizations and emerging evidence". In: ed. Hicks JA. The Experience of Meaning in life: Classical Perspectives, Emerging Themes, and Controversies. Dordrecht, Clay Routledge: Springer Netherlands (2013).

[32] Park CL Folkman S. Meaning in the context of stress and coping. Rev Gen Psychol. (1997) 1:115–44. doi: 10.1037/1089-2680.1.2.115

[33] Waters TE Shallcross JF Fivush R. The many facets of meaning making: comparing multiple measures of meaning making and their relations to psychological distress. Memory. (2013) 21:111–24. doi: 10.1080/09658211.2012.705300, 22900850

[34] Weber MC Hampton BN Schulenberg SE. Why is searching for meaning after trauma sometimes helpful and sometimes not? Adaptive coping as a moderator. Traumatology. (2025). doi: 10.1037/trm0000593

[35] Brady M. Can existentialist thinking about the meaning of adversity lead to post-traumatic growth? J Posit Psychol. (2025) 20:834–42. doi: 10.1080/17439760.2025.2505552

[36] Cameron EC Kalayjian A Toussaint L Cunningham FJ Jacquin KM. Meaning-making predicts forgiveness as an indicator of posttraumatic growth with a stronger effect for natural disasters. J Humanist Psychol. (2022) 66:335–59. doi: 10.1177/00221678221075910

[37] Dursun P Steger MF Bentele C Schulenberg SE. Meaning and posttraumatic growth among survivors of the September 2013 Colorado floods. J Clin Psychol. (2016) 72:1247–63. doi: 10.1002/jclp.22344, 27459242

[38] Yehuda R Hoge CW McFarlane AC Vermetten E Lanius RA Nievergelt CM . Post-traumatic stress disorder. Nat Rev Dis Prim. (2015) 1:1–22. doi: 10.1038/nrdp.2015.57 27189040

[39] Bonanno GA. Meaning making, adversity, and regulatory flexibility. Memory. (2013) 21:150–6. doi: 10.1080/09658211.2012.745572, 23311413 PMC3565080

[40] Park J Baumeister RF. Meaning in life and adjustment to daily stressors. J Posit Psychol. (2017) 12:333–41. doi: 10.1080/10720537.2015.1119082

[41] Frankl VE. The will to Meaning: Foundations and Applications of Logotherapy. Newyork: Penguin (2014).

[42] Yalom ID. Existential Psychotherapy. Newyork: Basic books (2020).

[43] Mutuyimana C Maercker A. How meaning in life and vitality are associated with posttrauma outcomes: a systematic review. J Trauma Stress. (2024) 37:551–62. doi: 10.1002/jts.23040, 38580621

